# Visual state estimation in unseen environments through domain adaptation and metric learning

**DOI:** 10.3389/frobt.2022.833173

**Published:** 2022-08-19

**Authors:** Püren Güler, Johannes A. Stork, Todor Stoyanov

**Affiliations:** Autonomous Mobile Manipulation Lab, Örebro University, Örebro, Sweden

**Keywords:** articulated pose estimation, joint state estimation, deep metric learning, domain augmentation, triplet loss

## Abstract

In robotics, deep learning models are used in many visual perception applications, including the tracking, detection and pose estimation of robotic manipulators. The state of the art methods however are conditioned on the availability of annotated training data, which may in practice be costly or even impossible to collect. Domain augmentation is one popular method to improve generalization to out-of-domain data by extending the training data set with predefined sources of variation, unrelated to the primary task. While this typically results in better performance on the target domain, it is not always clear that the trained models are capable to accurately separate the signals relevant to solving the task (e.g., appearance of an object of interest) from those associated with differences between the domains (e.g., lighting conditions). In this work we propose to improve the generalization capabilities of models trained with domain augmentation by formulating a secondary structured metric-space learning objective. We concentrate on one particularly challenging domain transfer task—visual state estimation for an articulated underground mining machine—and demonstrate the benefits of imposing structure on the encoding space. Our results indicate that the proposed method has the potential to transfer feature embeddings learned on the source domain, through a suitably designed augmentation procedure, and on to an unseen target domain.

## 1 Introduction

### 1.1 Motivation

In recent years, deep learning models have increasingly been applied to solve visual perception problems in robotics. For structured environments such as factories or warehouses that are not changing dramatically over time, training such models and obtaining successful results in test data is possible. However, for fully autonomous operations, these methods should work under test conditions in unstructured and unpredictable environments as well—e.g., in scenes with continuously changing background, illumination or appearance. Data augmentation is one common approach to enhancing the ability of deep visual models to cope with unexpected changes in the environment. The basic principle is to increase robustness by introducing synthetic changes to the source domain during training, such as changing background or texture, cropping images, introducing artificial camera noise. Yet, simply adding more samples to the training data may not be enough to cover every scenario that can occur during testing^1^.

To address the discrepancies between domains, models need to learn what are the task-relevant features in the data. Data augmentation helps to accomplish this by simply showing the model more varied data during training. However, there is an alternative: explicitly supervising what training samples should be considered similar or dissimilar by the model. Metric learning is one such alternative that aims to find an appropriate way to structure the similarities and differences in the underlying data (Kaya and Bilge (2019)). Metric learning however typically requires annotated data from all the potential target domains during training (e.g., detecting faces from different viewpoints (Schroff et al. (2015))). However, collecting and labeling sufficient data from all potential domains is at best time consuming and often impossible in a robotics scenario. In this work we explore the possibilities of combining the two approaches: domain data augmentation and metric learning. This allows us to use a metric learning objective without access to labeled data from the target domain, making a principled approach to domain augmentation possible.

**FIGURE 1:**
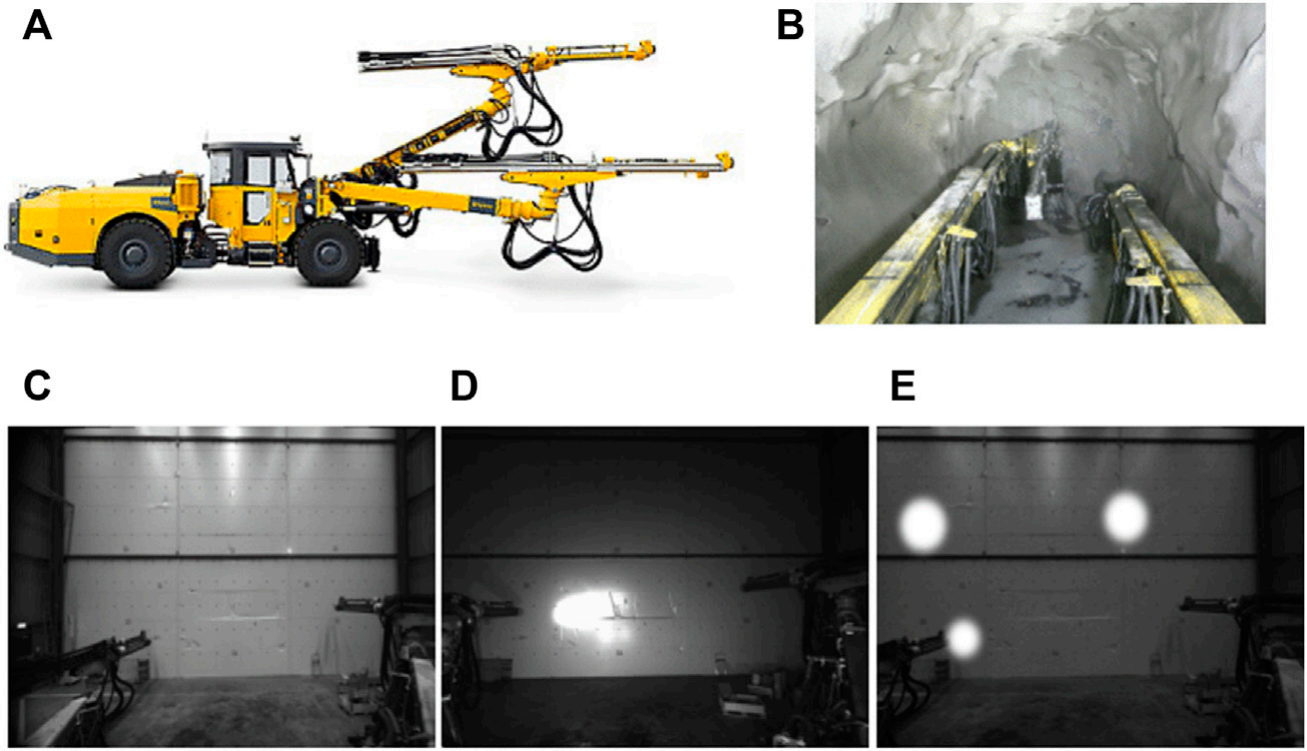
**(A)** A heavy-duty drilling machine with an articulated manipulator produced by the mining equipment manufacturer Epiroc ^1^ **(B)** Cabin view from the same machine operating in a mine **(C–E)** Epiroc testing warehouse. We collect a *source domain* data set with the hall lights on in **(C)**, as well as a *target domain* data set with only on-board lightning in **(D) (E)** shows augmented source domain data that we alter to simulate conditions in the target domain.

The target application we investigate in this work is the visual state estimation of an articulated mining machine (Figure 1A). Kinematic chains, such as traditional robot manipulators and the booms of our mining machine, are composed of individual links coupled with actuators. The state estimation problem is thus typically solved by measuring angles between links through joint encoder sensors. However, encoders can cause erroneous pose estimates due to sensor noise, cable strain, deflection or vibration of the manipulator. Drilling rigs that are used in mining and construction operate in dangerous and highly corrosive environments (Figure 1B). Hence, encoder sensors and data cables are subject to high wear and tear, motivating the need for a redundant visual state estimation system (Figures 1C–E).

### 1.2 Related work

Our work is at the intersection of several different field, i.e., robotics, computer vision, machine learning, and topics, e.g., transfer learning, domain augmentation, metric learning, triplet loss etc. To give a comprehensive overview for each of the related works from these topics is out of scope of this paper. In this section, we briefly overview each related topics very briefly and list the papers that we see the most relevant for our work.

#### 1.2.1 Robotics pose estimation through vision

In recent years, several studies in robotics have focused on estimating the pose of articulated links through visual sensors. Approaches based on markers (Vahrenkamp et al. (2008)), as well as on depth data and 3D models (Krainin et al. (2011); Klingensmith et al. (2013); Schmidt et al. (2014)) have been proposed. A large amount of work uses discriminative approaches that learn a direct mapping from the features of visual data (e.g., RGB or point cloud) to joint states or pose of articulated links (Widmaier et al. (2016); Byravan and Fox (2017); Zhou et al. (2019)). These features are usually extracted using either hand-made feature extractors or more end-to-end approaches such as Convolutional Neural Network (CNN) models. We choose the latter type of approach and employ a CNN architecture that can learn complex tasks directly from visual data (Krizhevsky et al. (2012)).

The feature-based methods mentioned above rely on the availability of a large amount of annotated data from both source and target domain. However, it may not be possible to collect annotated data for all the conditions a robot can encounter in a complex uncontrolled real-world environment such as an underground mine.

#### 1.2.2 Transfer learning

Transfer learning is a huge field that have been categorized in several ways, e.g., *label-setting* wise where labels of source and/or target domain are available (transductive, inductive) or unavailable (unsupervised), *domain feature space* wise where source and target domain feature spaces are similar (homogeneous) or different (heterogeneous), *field/topic wise* such as deep learning, computer vision, activity recognition etc. (Zhuang et al. (2020)). To give a detail analysis and comparison for each of these different types of categorizations with respect to our proposed method is out of scope of this paper.

However, in brief, the objective of transfer learning is to improve the generalization of a learned model on the target domain by transferring knowledge contained in different but related source domains. This objective is accomplished by minimizing the distance between target and source domain data during training (e.g., Ganin et al. (2016); Tzeng et al. (2017); Laradji and Babanezhad (2020)). This naturally requires access to target domain data during training or fine-tuning, which as mentioned previously is often not readily available. Differently, in our work, we apply domain-aware augmentation to the source domain data without requiring training/fine-tuning in target domain.

#### 1.2.3 Domain augmentation

Domain augmentation is a way of overcoming the data scarcity problem by adding a large amount of annotated synthetic data or by transforming existing data. Data augmentation is a huge field (e.g., Shorten and Khoshgoftaar (2019)) with various techniques and in-depth discussion of each of these techniques is out of scope of this paper. Nevertheless, we can say that the techniques such as background augmentation, adding noise or cropping/transforming images, are common means to increasing the data variation in the source domain (Lambrecht and Kästner (2019); Gulde et al. (2019); Lee et al. (2020); Labbe et al. (2021)). The model is then trained under more varied conditions which helps improve generalization and break the dependence on annotated data from the target domain. In our work, rather than such random augmentations, e.g., random noise injection in images or geometric transformations, we apply a domain-aware augmentation by assuming target domain knowledge is available. Hence, even though we do not have sufficient target data, we complete this insufficiency through target domain-aware augmentation of source data.

#### 1.2.4 Metric learning

Metric learning is another approach to improving model generalization by learning the relation between samples in a dataset belonging to a certain domain. Learning such relations imposes a structure to the feature encoding domain, which in turn has been demonstrated to improve transfer in various applications, such as multi-view face recognition (Schroff et al. (2015)), medical imaging (Litjens et al. (2017)) or remote sensing for hyperspectral image classification (Dong et al. (2021)). The main challenges when combining deep learning with metric learning include the design of the metric loss function (e.g., contrastive or triplet loss function), the strategy for selecting samples (e.g., hard-negative, semi-hard negative), and the design of the network structure (e.g., siamese, triplet networks) (Kaya and Bilge (2019)). We apply a standard triplet loss and propose a domain-specific sample selection strategy as our contribution.

### 1.3 Problem definition and contribution

In this article we aim to address some of the challenges in transferring learned vision-based models to new domains. In particular, we are interested in training a machine learning model for operation in an environment in which we are not able to collect data. We instead propose to use the background knowledge and prior information available at design time in order to appropriately augment the training procedure.

In doing so, our contributions are as follows:

• We combine techniques from domain augmentation—namely, the use of a designed augmentation procedure—and from metric learning.
• We adapt the triplet learning methodology and propose an approach for principled integration of domain-augmented data as a source for both positive and negative examples. Our main contribution is thus the said principled treatment of domain augmentation with the purpose of transfer of a vision-based learned model.
• We evaluate our approach on a data set within mining robotics, thus demonstrating the practical use of the proposed approach.

## 2 Methods

In this section we present a learning architecture aimed at recovering the joint angles of an articulated kinematic chain from visual observations. We design our approach to utilize domain adapted training data to improve model transfer to images collected in previously unseen environments. We accomplish this by posing two objectives—a primary joint recovery objective and a secondary metric learning objective. This section begins with a problem specification in Section 2.1, followed by a discussion of the base joint regression task in Section 2.2. Next, in Sections 2.3, Section 2.4, Section 2.5. We augment our method with a secondary objective that aims to learn a smooth feature embedding space.

### 2.1 Learning a generalizable visual model

In this paper we are interested in solving a particular task relevant to mining robots: the visual state estimation problem. The base problem of recovering the robot state from visual observations has been previously discussed in other contexts, such as e.g. for robot manipulators (Zhou et al. (2019)). Given sufficient observations, it is possible to successfully train a neural network architecture, such as the one described in the following section. The challenge here lies in the difficulty of collecting sufficiently varied observations that span the full range of possible operating conditions for the machine. This problem is often solved via data augmentation, but as we show here, data augmentation alone may not be sufficient to guarantee good transfer of the learned visual models to out-of-domain data.

We formalize our problem as follows. We assume access to a sufficiently large data set of in-domain annotated examples. In our case these are supervised pairs of images **I** and measured robot joint configurations **q** from an onboard encoder system. In addition, we assume some prior knowledge of the target domain, which allows us to design an imperfect, yet admissible data augmentation procedure *g*_*aug*_(**I**). The goal is then to best use the fixed data augmentation procedure in order to train a model that successfully generalizes to a novel domain.

### 2.2 Regressing joint states

**FIGURE 2:**
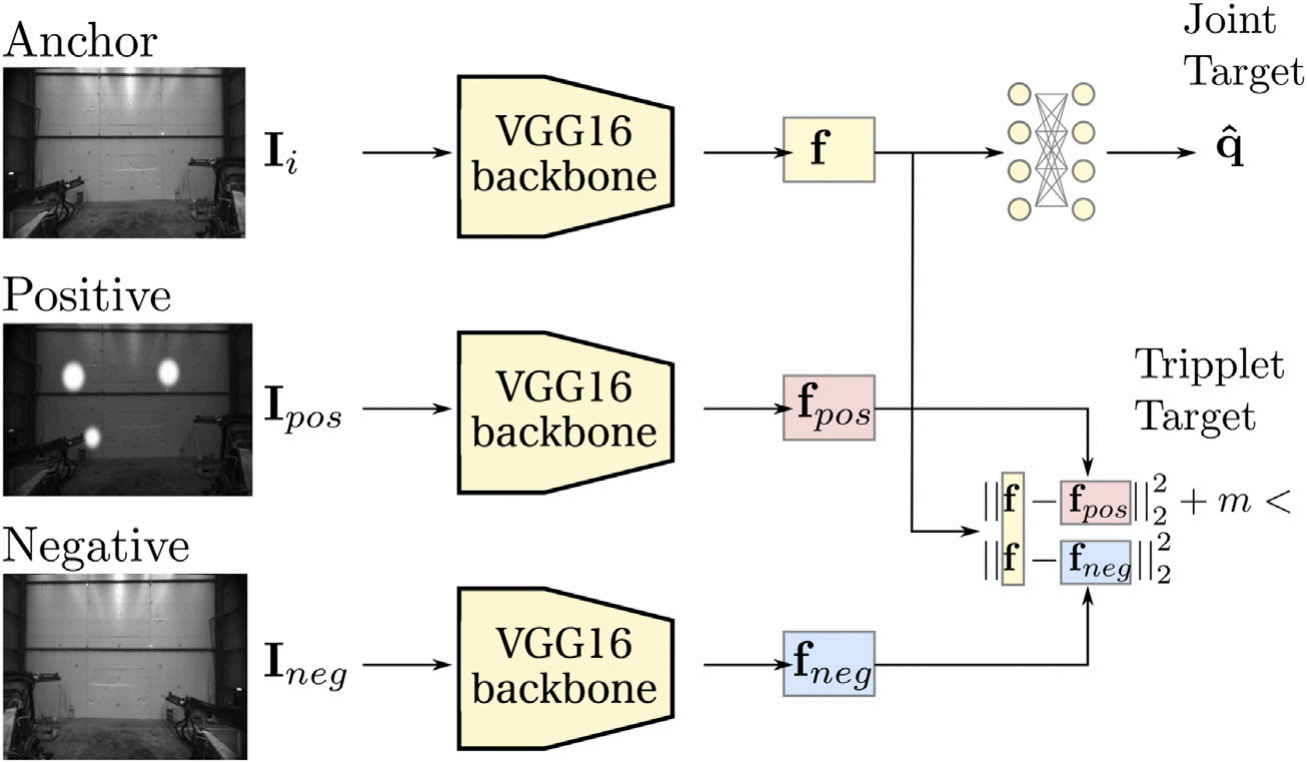
The overall proposed machine learning architecture. We use a CNN model based on the VGG16 architecture as a backbone for feature extraction. We extract feature embeddings **f** from a given image **I** and regress the joint state **q** via a fully connected output layer. In addition, we pose a metric learning objective where we strive to keep the embedding **f** close to select positive examples (**f**_*pos*_) and far from select negative ones (**f**_*neg*_).

Our approach is based on a CNN that extracts feature embeddings **f**, given a batch of RGB images **I**. The CNN is trained on a *source* domain of images, where each sample depicts a predetermined articulated kinematic chain (e.g., manipulator, machine boom) in a known configuration **q**. The joint regression task is thus to estimate a configuration $\hat{q}$ that is as close as possible to the true configuration **q**. We use the VGG16 network architecture (Simonyan and Zisserman (2014)) as a backbone for the feature extraction task and initialize it using weights pre-trained on the ImageNet classification data set (Deng et al. (2009)). Note however the proposed method is not dependent on any single CNN backbone and VGG16 could be substituted by an alternate feature extraction architecture. We then supervise the feature extraction task with a joint regression head, as seen in Figure 2 and outlined below.

The backbone, based on the VGG16 architecture (Simonyan and Zisserman (2014)), feeds the input image **I**_*i*_ through a series of convolution layers. We use all convolutional and pooling layers of VGG16, but discard the last fully connected layers, i.e., *FC-4096* and *FC-1000* in (Simonyan and Zisserman (2014)). Hence, the last layer of the backbone is the fifth maxpool layer of VGG16 and **f** is the feature embedding extracted from this maxpool layer. We regress the joint target $\hat{q}$ in Figure 2 via two fully-connected layers, ***fc***. These layers have the same input structure as the *FC-4096* layer of VGG16 and for that reason we resize the input image to 224 × 224 using nearest-neighbor interpolation.

We supervise the joint target regression task with a loss defined on the predicted state $\hat{q}$ . In our evaluation scenario discussed in Section 3.1 we have an output space with $q\in R^{7}$ , where each dimension represents the state of a joint in the kinematic chain. Five of these joints are revolute, while two are prismatic, resulting in a non-homogeneous configuration vector which is partially defined in radian and partially in meters. To counter to this difference, we regress radian $\left({\hat{q}}_{\mathrm{rad}}\in R^{5}\right)$ and meter joint states $\left({\hat{q}}_{\mathrm{met}}\in R^{2}\right)$ in different layers simultaneously. The range of motion of joints in radian can be between 0 and 2*π*. Hence, to avoid issues due to angle wraparound, we define our regression loss function over a cosine/sine transform of the radian joint angles and concatenate them in a single array, $\hat{q}=\left\{\cos\left({\hat{q}}_{\mathrm{rad}}\right),\sin\left({\hat{q}}_{\mathrm{rad}}\right),{\hat{q}}_{\mathrm{met}}\left.\;\right)\right\}\in R^{12}$ . Then, the loss for a batch of size *n*_*batch*_ is calculated by computing the Mean Squared Error (MSE) between the ground-truth $q\in R^{n_{\mathrm{batch}}\times 12}$ and estimated $\hat{q}\in R^{n_{\mathrm{batch}}\times 12}$ :

$$L_{js}=\frac{1}{n_{\mathrm{batch}}\times 12}\overset{n_{\mathrm{batch}}}{\underset{j=1}{\sum }}\overset{12}{\underset{i=1}{\sum }}{\left(q_{i,j}-{\hat{q}}_{i,j}\right)}^{2}$$ (1)

### 2.3 Learning a metric space

Estimating joint states from visual input, as described in the previous section, works well if we have sufficient in-domain data. In this work we are however interested in a case when such data are not readily available. To improve our model’s generalization potential we lean on the concept of metric space learning. In particular, we employ a triplet loss function similar to the ones used in (Schroff et al. (2015); Sun et al. (2014)). It is a well known loss function. However, for the sake of completeness of the methodology, we explain the details of our usage of triplet loss function in this section.

Given an image **I**_*i*_, we aim to extract a lower-dimensional feature embedding $f\in R^{d}$ . Intuitively, we want that our embedding should map similar images to close by feature vectors, while dissimilar images should map to locations that are far apart. Crucially, similar in this case signifies a similarity in terms of the primary task—that is, images that show the articulated manipulator chain in close by configurations—and not in terms of image similarity per say. We bias our model to learn such an embedding by feeding the network with a triplet of images—associating to every sample **I**_*i*_ a similar image **I**_*pos*_ and a dissimilar image **I**_*neg*_—as seen in Figure 2. In the metric learning literature, these images are known as the *anchor* **I**_*i*_, the *positive* **I**_*pos*_ and the *negative* **I**_*neg*_.

As depicted in Figure 2, the three images are embedded to corresponding feature-space vectors via copies of our backbone architecture, where the weights of the three networks are shared. The corresponding feature embeddings **f**, **f**_*pos*_ and **f**_*neg*_ are extracted from the final fully connected layer of the backbone networks and normalized. We want to enforce a margin *m* between similar and dissimilar features where:

$${\left\|f-f_{\mathrm{pos}}\right\|}_{2}^{2}+m<{\left\|f-f_{\mathrm{neg}}\right\|}_{2}^{2}$$ (2)

Hence, we formulate and minimize the following loss (Triplet Target in Figure 2):

$$L_{\mathrm{triplet}}=\overset{n_{\mathrm{batch}}}{\underset{i}{\sum }}\max\left(\|f^{i}-{f^{i}}_{\mathrm{pos}}{\|}_{2}^{2}-{\left\|f^{i}-{f^{i}}_{\mathrm{neg}}\right\|}_{2}^{2}+m,0\right)$$ (3)

where **f** ^*i*^ is *i*th element in the batch. We incorporate this secondary objective in the overall training loss, which corresponds to our modification in usage of the triplet loss function. It is minimized as:

$$L_{\mathrm{total}}=wL_{js}+\left(1-w\right)L_{\mathrm{triplet}}$$ (4)

where *w* is a weight specifying the relative importance between the primary (*L*_*js*_) and secondary (*L*_*triplet*_) targets.

### 2.4 Selecting samples

Choosing the negative and positive examples to use for each anchor in a triplet is known to be critically important for fast convergence and good performance. Hence finding anchor-negative pairs that violate Equation 2 (i.e., hard-negatives) is important (Schroff et al. (2015)). To select negatives, we use an online negative exemplar mining strategy from the whole training data set. In this section, we explain our proposed online negative mining strategy adapted for our dataset.

At the end of each training epoch, we calculate and store the Euclidean distance between the embedded features of each training sample, obtaining a confusion matrix *C*_*f*_(**f**) ∈ *R* ^*N*×*N*^ (where *N* is the cardinality of the training data set):

$$C_{f}\left(i,j\right)=\|f^{i}-f^{j}{\|}_{2}$$ (5)

In addition, we also calculate and store the distance between ground-truth joint state of each training sample, obtaining another confusion matrix *C*_*q*_(**q**) ∈ *R* ^*N*×*N*^:

$$C_{q}\left(i,j\right)=\|q_{i}-q_{j}{\|}_{2}$$ (6)

Then, for each sample *i*, we eliminate all samples *k* that are too close in terms of joint configuration, that is *∀k*: *C*_*q*_ (*i*, *k*) < *α* with a preset similarity threshold *α*. Finally, we select hard-negative samples among the remaining possible pairs by looking up the feature-space confusion matrix *C*_*f*_ and choosing the closest feature-space sample $\underset{r}{\arg\;\min}C_{f}\left(i,r\right)$ .

### 2.5 Data augmentation

We apply the domain-aware augmentation procedure *g*_*aug*_ randomly with 50% chance to the negatives **I**_*neg*_ mined from the source domain. This results in negative images that are appearance-wise both dissimilar (*g*_*aug*_ (**I**_*neg*_)) and similar (**I**_*neg*_) to the anchors **I**_*i*_. For positive pair selection, we apply augmentation to each anchor image **I**_*pos*_ = *g*_*aug*_ (**I**_*i*_) and select it as the positive pair for anchor **I**_*i*_. Augmentation makes positive images appearance-wise dissimilar to the anchor image, while keeping an identical joint state configuration. Hence intuitively, we aim to bring closer the embeddings of these visually distinct images by learning to abstract from appearance and focus on what matters for the primary task.

## 3 Materials and equipment

In this section, we overview our data collection and experimental setup in Sections 3.1 and Section 3.2.

### 3.1 Dataset collection

**FIGURE 3:**
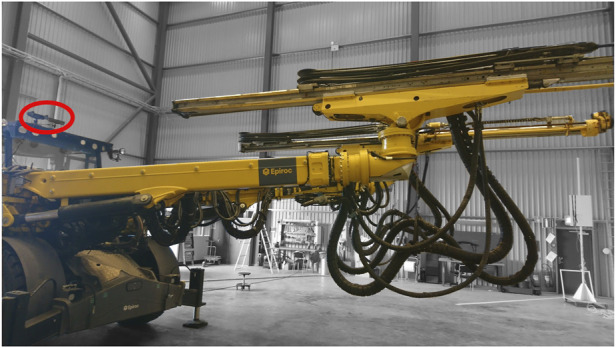
We mount a MultiSense S21 stereo camera on the operator cabin for data collection. Sensor placement is indicated by red oval in figure. Alternate mounting locations were explored in different data collection runs.

We evaluate our approach on a task of visual state estimation for a drilling rig (see Figure 3). The input of our method is an RGB image, **I** ∈ *R* ^224*x*224*x*3^, while the expected outputs are the joint configurations **q** describing the state of one articulated boom of the machine. We measure **q** by means of a number of encoder and resolver modules attached to each rotational and prismatic joint of the boom and connected to the vehicle’s CAN network. Simultaneously, we record corresponding images from a MultiSense S21 stereo camera, mounted on top of the operator cabin as shown in Figure 3. Hence, we train the network in our method using **I** as input, with the ground-truth joint angle states **q** as output targets. Collecting simultaneously the ground-truth joint angle states through CAN network and the images from the stereo camera is implemented via the Robot Operating System (ROS) (Quigley et al. (2009)). We conducted our experiments on a computer with GeForce RTX 2080 as GPU and an Intel(R) Xeon(R) E-2176G CPU.

We collect data under two different sets of conditions, mimicking the scenario that our system would need to face in a real deployment. The machine is deployed in a service hall and we record images of the boom in different configurations. We do so first with the hall lights on, creating a *source domain* data set with good lightning conditions (Figure 1C). Next, we repeat the data acquisition but with the hall lights switched off and the on-vehicle headlights turned on, creating a second *target domain* data set (Figure 1D). This setup is meant to mimic the real deployment conditions of our system, wherein it is not possible to collect data from all target domains likely to occur in the field.

Overall, our data set consists of 20,066 annotated images from the source domain, and 6,693 corresponding images in the target domain. In both cases the range of motions of the booms observed in the two data sets are similar. We partition the data sets in a 60/20/20 split for training, validation and testing. We apply the augmentation procedure *g*_*aug*_ only to the source domain data. In our experiments, *g*_*aug*_ involves adding a randomly weighted Gaussian noise to each pixel, randomly decreasing the brightness of the full image with up to 40%, and randomly adding simulated specular reflections. The last step is meant to replicate the oversaturated reflections of the vehicle headlights in the target domain and is implemented by superimposing random white circles of varying radius and with edges smoothed by a Gaussian filter (example shown in Figure 1E).

### 3.2 Training details

We use TensorFlow’s estimator API for implementing our network architecture. The joint regression loss *L*_*js*_ is calculated for each batch with size *n*_*batch*_ = 8 as described in Section 2.2. For calculating the triplet loss *L*_*triplet*_ we use batches of *n*_*batch*_ = 8 image triplets **I**, **I**_*pos*_ and **I**_*neg*_. The triplet loss metaparameter *m* in equation 3 is set to 0.05. We combine the two losses using equation 4, with *w* set to 0.1. Finally, the metaparameter *α* used in mining of negatives (see Section 2.4) is set to 0.25.

We use the Adam optimizer (Kingma and Ba (2014)) to minimize the total loss and train the network end-to-end. Adam is a broadly used adaptive optimization algorithm for deep learning applications in computer vision. It is a fast converging optimization algorithm. Triplet loss is a difficult loss function where speed of convergence can slow down e.g., due to sample selection. Hence, we want to use an optimizer that can speed up the convergence process. We expect that its estimation quality should be comparable with other optimizers used in deep neural networks. Therefore, we use it due to its being a fast converging and common practice method in deep learning field. We set Adam’s learning rate of 1e-5 and apply early stopping. We set *λ* and dropout to 5e-4 and 0.5 respectively for regularization. In addition, L2 regularization is applied in each layer and dropout is applied in the final fully connected (***fc***) layers. We apply early stopping by terminating training if the loss does not decrease for three consecutive epochs in the validation set.

We distinguish five distinct training/testing conditions. In all cases we evaluate the trained architectures on the retained test data from the *target* domain.

• Baseline target (BT): As a baseline we train a version of our architecture that only contains the joint state estimation head—that is, optimizing only the loss *L*_*js*_. The baseline is given access to the training set from the target domain and represents the *ideal* case. That is, the best possible performance achievable by the architecture, if sufficient labeled in-domain data were available. We note that this baseline should not be taken as the performance we aim to achieve, since the premise of this work is that we operate in a regime in which it is not possible to collect data from all conceivable deployment domains.
• Pre-trained baseline source (PBS): Under this condition we directly transfer a network trained on the source domain and evaluate it on the target domain. This case represents the naive approach of hoping for the best and is meant to evaluate the difficulty of generalizing between our two domains.
• Pre-trained source domain data with 12k data augmentation (PDA12k): It is a network trained only on the joint estimation task, using source domain data that is augmented with an dditional 12k samples (i.e., doubling the training data by providing one augmented sample for each).
• Pre-trained triplet loss with source (PTrip): It represents the proposed approach. We train using both the joint state estimation and metric learning losses, where we use the same data as in the previous condition—all source domain training data, plus an additional 12k augmented images.

## 4 Results

### 4.1 Estimation accuracy

As a first step, we evaluate the different transfer approaches based on the primary task error. To evaluate the joint state estimation error, we extract the estimates ${\hat{q}}_{\mathrm{rad}}$ and ${\hat{q}}_{\mathrm{met}}$ separately from each architecture. Then, as explained in Section 2.2, to avoid angle wrap-around errors we apply the cosine/sine transform to the rotational joints. The transformed radian joint states and meter joint states are concatenated in $\hat{q}\in R^{12}$ . For each data sample *i* ∈ *N*, where *N* is number of test data, the prediction error is calculated as the $L_{2}$ norm:

$$Err_{js}=\|q_{i}-{\hat{q}}_{i}{\|}_{2}$$ (7)

Since the error distribution is not Gaussian, rather than mean and standard derivation over the whole test data set, we compare the median and interquartile range (IQR).

According to Table 1, both PDA12K and PTrip decrease the error significantly, compared to direct transfer (PBS). Hence, our way of using data augmentation with a triplet loss increases the transferability capacity of the baseline model trained only on source domain. However, even with the best performance, the error of prediction with the transferred models is still much higher (≈7 times) than the BT model which is trained directly on labeled target domain data.

**TABLE 1:** Median joint-space error *Err*_*js*_. The bold text indicates the best results among transferred models (PBS, PDA12K, PTrip).

	BT	PBS	PDA12k	PTrip
**Median**	*0.0937*	0.91	**0.639**	0.71
**IQR**	*0.0931*	0.53	0.567	**0.435**

We also test the prediction accuracy of the evaluated models on a secondary task—that is, the task of pose estimation for the links of the boom. In reality, this secondary task is of more interest in our application, but is very challenging to efficiently supervise the network. We calculate the error of pose estimation of the end-effector using the model-based displacement measure (DISP) introduced by (Zhang et al. (2007)). DISP calculates the maximum distance between corresponding vertices of a mesh model of a given manipulator, when placed in different configurations. In our case we are interested in the DISP measure between the ground-truth configuration **q** and the estimated configuration $\hat{q}$ . This measure provides a more interpretable metric and directly correlates with the expected accuracy in task space when using the estimated joint configurations. Formally, we calculate the measure over all points *p* that are vertices of the manipulator mesh *M* as:

$$DISP_{M}\left(q,\hat{q}\right)=\underset{p\in M}{\max}\|p\left(q\right)-p\left(\hat{q}\right){\|}_{2}$$ (8)

where **p**(**q**) is the position of point **p** when the model is placed in joint configuration **q**.

The DISP errors for each evaluated approach are shown in Table 2. We note that our proposed approach with domain-aware data augmentation and triplet selection performs best at this measure. Both our full approach and the domain-aware data augmentation variant result in improved pose estimation, compared to the direct transfer approach. Overall, the PTrip approach results in an improvement of roughly 30% compared to the direct transfer baseline (PBS). While this is encouraging, we note that all transfer approaches remain far from the desired performance attained by the method trained in-domain.

**TABLE 2:** Median DISP error that calculates error of the pose of end-effector in meter for target domain data with different training/testing approaches. The bold text indicates the best results among transferred models (PBS, PDA12K, PTrip).

	BT	PBS	PDA12k	PTrip
**Median**	*0.265*	1.719	1.487	**1.198**
**IQR**	*0.189*	1.046	0.819	**0.67**

**FIGURE 4:**
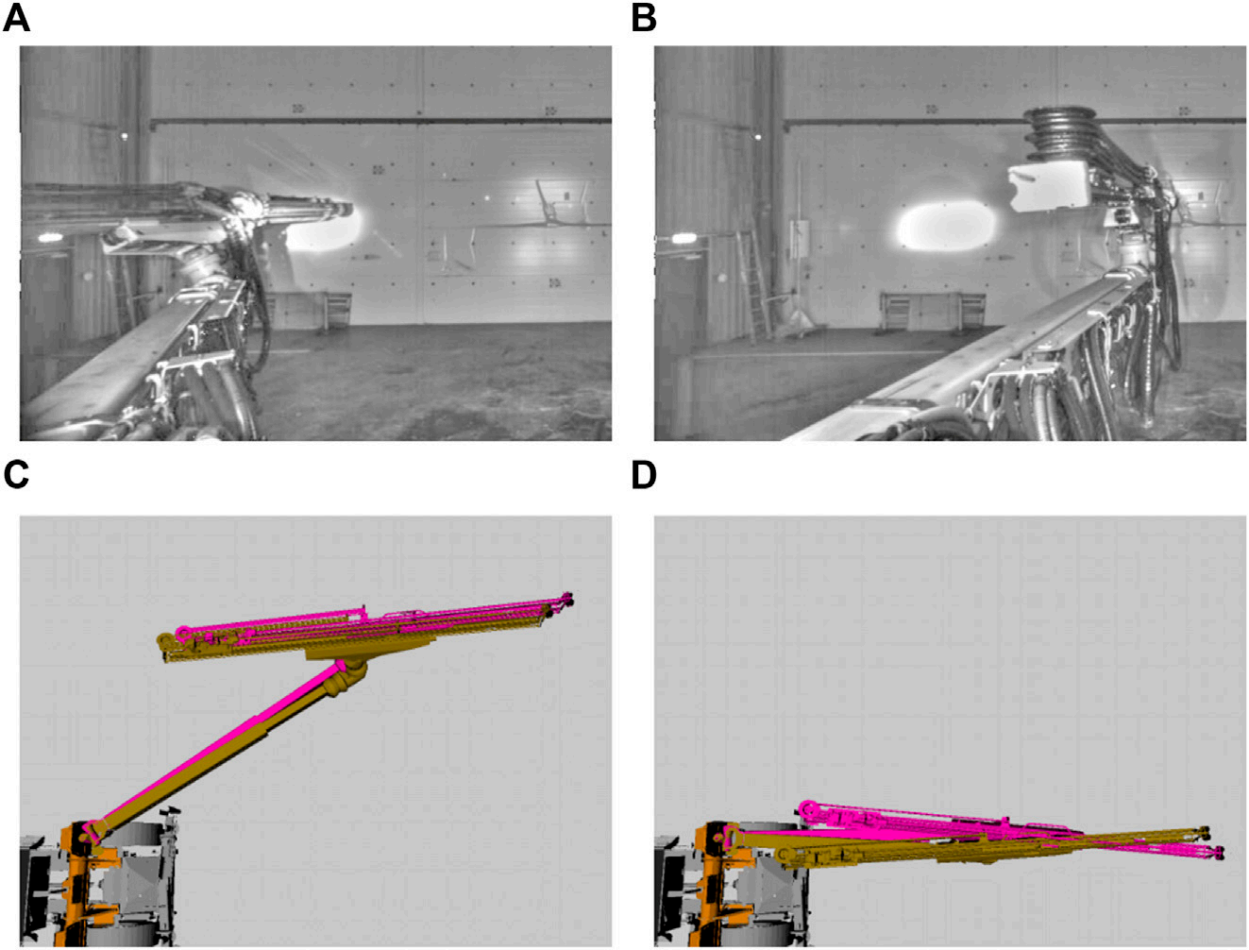
**(A)**, **(B)** Two example frames from the target data set. Contrast and colors re-adjusted for clarity of display **(C)**, **(D)** Corresponding ground truth (in yellow) and predicted (in purple) boom configurations. The prediction in **(C)** results in a large joint state error, but a low DISP measure (*Err*_*js*_ = 0.6, DISP = 0.57). On the other hand, the prediction in **(D)** results in a low JSE, but a high DISP measure (*Err*_*js*_ = 0.35, DISP = 0.89).

It is important to note that, although our approach performs better on the DISP measure, we did not directly supervise this task, and consequently there is a degree of randomness to this outcome. Our intuition is that the metric space learning objective forces our prediction model to make errors in a similar direction for similar joints. The results is that, although PTrip often makes errors in predicting a joint configuration comparable to those of the PDA12K model, these errors are correlated and often cancel out. As an illustrative example, consider Figure 4. Two input images are shown, along with a corresponding birds’s-eye view visualization of the estimated and ground-truth configurations (Figures 4A,B). The prediction in Figure 4C has a higher configuration-space error than the one in Figure 4D. However, the bulk of the error in the first case is distributed on the two prismatic axes, with opposite error magnitude. This results in a lower DISP measure for the estimate in Figure 4C. Visually, this result is not unexpected, as the models make predictions based on appearance, and in appearance space the two predictions in Figure 4C are much closer. We note this unexpected benefit of our proposed method and defer deeper investigation for future work.

### 4.2 Statistical analysis

To determine whether the error results of transfer learning models stated in Table 1 and Table 2 are not random and their difference are statistically significant, we apply further statistical tests. We chose Mood’s median test (Mood (1954)) to do that due to the error distributions are non-Gaussian, as we state above. Mood’s median test is a non-parametric statistical test. It can replace more common statistical tests such as *t*-test or ANNOVA that requires normal data assumption. Hence, we use Mood’s median test to show that the accuracy results we state in Table 1 and Table 2 are not randomly found values but they have statistical significance.

Mood’s median is a very well known statistical test. Yet, for the sake of completion, we explain it very briefly. The null hypothesis of Mood’s median test is that the population medians are all equal, hence there is no significant difference between populations. To assess this null hypothesis, we choose *α* = 0.05 as significance level. Then, to test the null hypothesis, a chi-square value is calculated between *k* populations. In our case, we compare PBS, PDA12K and PTrip’s error results with each other two by two, i.e., *k* = 2. Another important parameter is critical value that we compare the calculated chi-square value with. If chi-square is bigger than the critical value, we can reject the null hypothesis and the difference between our errors states in Table 1 and Table 2 are meaningful. The critical value is determined based on *k* and chosen *α*. For *k* = 2 and *α* = 0.05, the critical value is determined as 3.841.

For clarity, we show the chi-square results in a matrix format in Table 3 and Table 4. Our results clearly show that all the chi-square values are at least 10 times bigger than critical value in Table 3 and Table 4. Hence, the null hypothesis is rejected and we can say that the difference between errors stated in Table 1 and Table 2 are statistically significant.

**TABLE 3:** Mood’s median test’s chi-square values calculated from joint state errors of different training/testing conditions. The italic ones shows the smallest chi-square values. The bold text indicates the best results among transferred models (PBS, PDA12K, PTrip).

	PBS	PDA12k	PTrip
**PBS**	N/A	156.912	157.15
**PDA12k**	156.912	N/A	*11.593*
**PTrip**	157.15	*11.593*	N/A

**TABLE 4:** Mood’s median test’s chi-square values calculated from DISP errors of different training/testing conditions. The italic ones shows the smallest chi-square values. The bold text indicates the best results among transferred models (PBS, PDA12K, PTrip).

	PBS	PDA12k	PTrip
**PBS**	N/A	*38.804*	226.528
**PDA12k**	*38.804*	N/A	135.63
**PTrip**	226.528	135.63	N/A

Also, the statistical results are consistent with the error results. For instance, the smallest chi-square value is between PTrip and PDA12k in Table 3. Therefore, the significance of difference between the errors of PTrip and PDA12k is not as high as the ones of PTrip and PBS, or, PBS and PDA12k. This results is consistent with the smallest error difference between PTrip and PDA12k, as shown in Table 1. We can observe similar consistencies between Table 4 and Table 2 for PBS and PDA12k as well.

### 4.3 Latent space analysis

**FIGURE 5:**
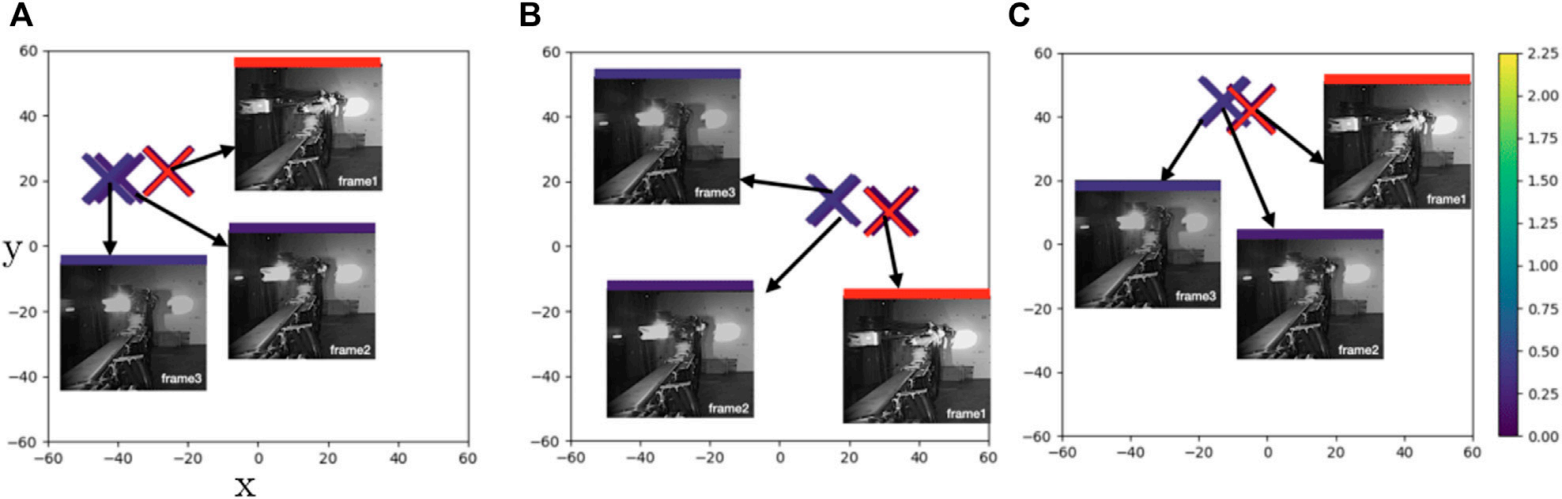
**Case1**: t-SNE plots of learned feature embeddings—**(A)** through PBS **(B)** PDA12K and **(C)** PTrip—colored with ground-truth joint states distance to a reference frame (red cross). Both PDA12K and PBD separates the points in similar distance while PTrip brings them closer together.

In addition to evaluating the primary task, we also analyze the performance according to our secondary metric learning objective. In particular, we are interested in the generalization properties of the learned feature encoders, and thus in this section we base our evaluation on sequences of images from the target domain. We embed both consecutive images with similar appearance and joint configuration, as well as images from remote sections of the data set. In order to visualize the obtained embeddings $f\in R^{d}$ , we map the whole target data set through each of the three test conditions PBS, PDA12K and PTrip. We then take the corresponding data sets of feature embeddings in *d* − dimensional space and pass them through another dimensionality reduction step to obtain an interpretable 2D visualization. For the latter step we use the popular t-SNE dimensionality reduction schema (Van der Maaten and Hinton (2008)), as it creates locally smooth embeddings relevant for each feature space. In this manner, we can easily discern how closely similar/dissimilar feature points place in the learned latent space (e.g., Figure 5) and qualitatively evaluate how well each approach captures the smoothness and structure of the target domain.

For clarity, we select several data points from some exemplary cases rather than plotting the whole feature space. To display the similarity in the primary task space we color the feature embeddings by Euclidean distance to a fixed reference configuration. We plot the embedding of the reference with a red cross (e.g., Figure 5) and use the same color scale in all images with lighter colors representing more dissimilar joint configurations. The euclidean distance is calculated using the cosine/sine transformed $\hat{q}\in R^{12}$ as explained in Section 2. We plot both the feature embedding, as well as the corresponding input.

Figure 5 illustrates the feature embeddings for a sequence of images that capture a yawing motion of the boom of the machine (**Case1**). Even though frame 2 is in almost equal distance to frame 1 and frame 3, it is placed closer to frame3 in both PBS (Figure 5A) and PData12k (Figure 5B). On the other hand, PTrip manages to bring them closer (Figure 5C) and thus results in a more faithful representation of these points in latent space. To verify this observation, we compare smoothness of the estimated joint configurations with smoothness of the ground-truth joint configurations. Smoothness of joint configuration is an important factor for robots to move end-effector accurately and in a smooth continuous manner along a specified trajectory. Therefore, we calculate the trajectory smoothness for the joint configurations ${\hat{q}}_{i}$ predicted by each model over the entire yawing motion. We measure smoothness using the center line average (CLA) metric: i.e., $\frac{1}{d}\frac{1}{n}\sum_{i}^{n}|{\hat{q}}_{i}-\bar{q}|$ where $\bar{q}$ is the sample mean and *d* = 12 We calculate the average CLA over normalized joint state estimates between 0 and 1 and report results in Table 5. We note for example that for **Case1** PTrip achieves a trajectory with comparable smoothness to the one featured by the ground-truth trajectory. Hence PTrip achieves the best representation of the ground-truth joint states in latent space by bringing similar features closer and keeping dissimilar ones apart.

**TABLE 5:** Center line average of predicted joint states for the selected cases discussed. The bold text indicates the best results among transferred models (PBS, PDA12K, PTrip).

	PBS	PDA12k	PTrip	Ground-truth
**Case1**	0.061	0.054	**0.038**	*0.020*
**Case2**	0.060	0.077	**0.069**	*0.070*
**Case3**	0.081	**0.072**	0.096	*0.055*
**Case4**	**0.041**	0.044	**0.041**	*0.024*

**FIGURE 6:**
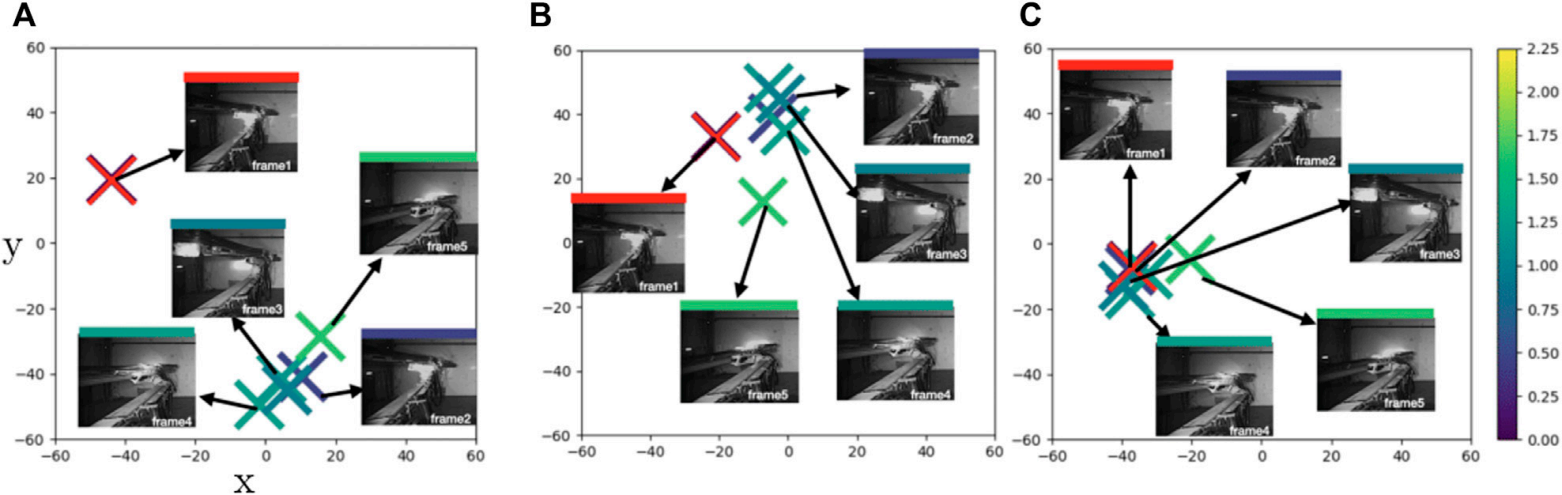
**Case2**: t-SNE plots of learned feature embeddings—**(A)** through PBS **(B)** PDA12K and **(C)** PTrip—colored with ground-truth joint states distance to a reference frame (red cross). PTrip brings similar blue dots closer than PBS and PDA12K.

In Figure 6, we display a more complex case (**Case2**) where the boom is executing a combination of motions of several joints simultaneously—i.e., the end effector is yawing, rolling and translating. In Figure 6A, PBS pushes frame 2 (dark blue) away from the reference point frame 1 (reference), while it brings frame 5 (light green) closer to frame 2. This creates an inconsistency. PDA brings these similar points, frame 2 and frame 1 closer while pushing frame 5 (a dissimilar point) further away (Figure 6B). However, the mild green frames such as frame4 that has almost the same distance to both frame 1 and frame 5 are pulled in closer to the darker points. Finally, PTrip finds a balance between these similar and dissimilar points (Figure 6C).

**FIGURE 7:**
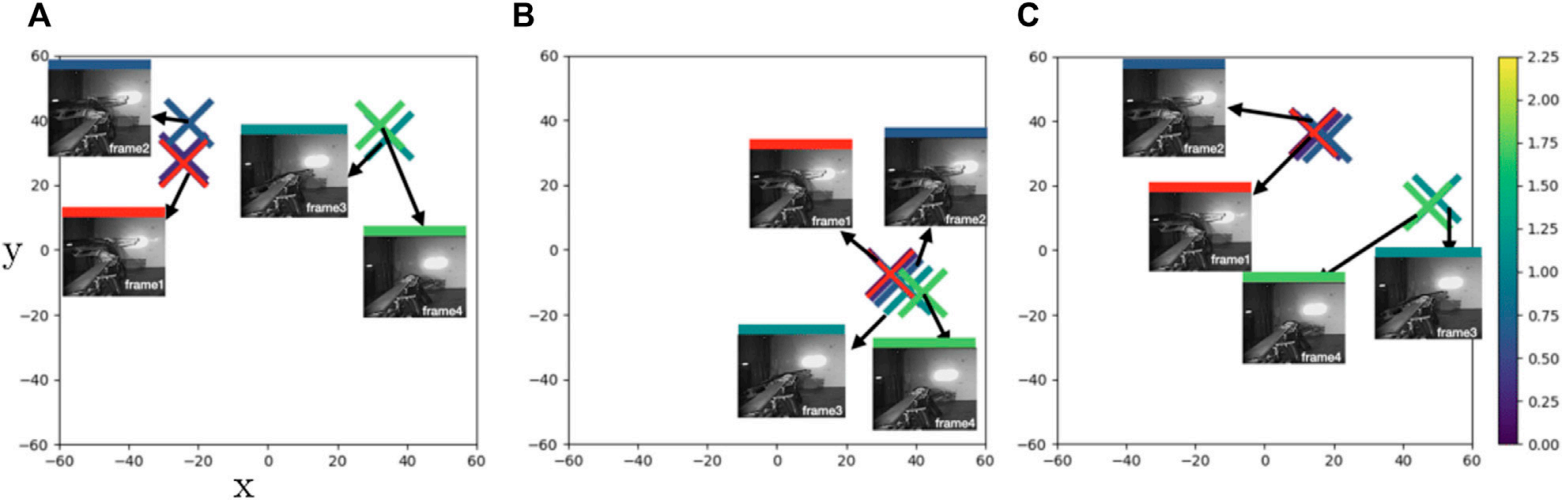
**Case3**: t-SNE plots of learned feature embeddings—**(A)** through PBS **(B)** PDA12K and **(C)** PTrip—colored with ground-truth joint states distance to a reference frame (red cross). PDA12K successfully brings similar points closer and provides a smooth transition between consecutive frames. PTrip fails to do so. But as we see **(A)**, ground truth data is also erroneous hence PTrip fails to correct the error. With a more accurate labeling for training, PTrip should do fine as well.

In **Case1** and **Case2** we examine sample motion sequences where PTrip performs better relative to the other models. However, there are cases where PTrip also fails in bringing/separating similar/dissimilar points in latent space. For instance, in Figure 7, frame 3 (mild green) is in equal distance to frame 2 and frame 4. But both PBS and PTrip place it in a closer place to frame 4 (Figures 7A,C), while PDA12k manages to place them in a more balanced way (Figure 7B). This reflects to the smoothness measure and PDA12k gives the closest average CLA to the ground-truth value (Table 5).

**FIGURE 8:**
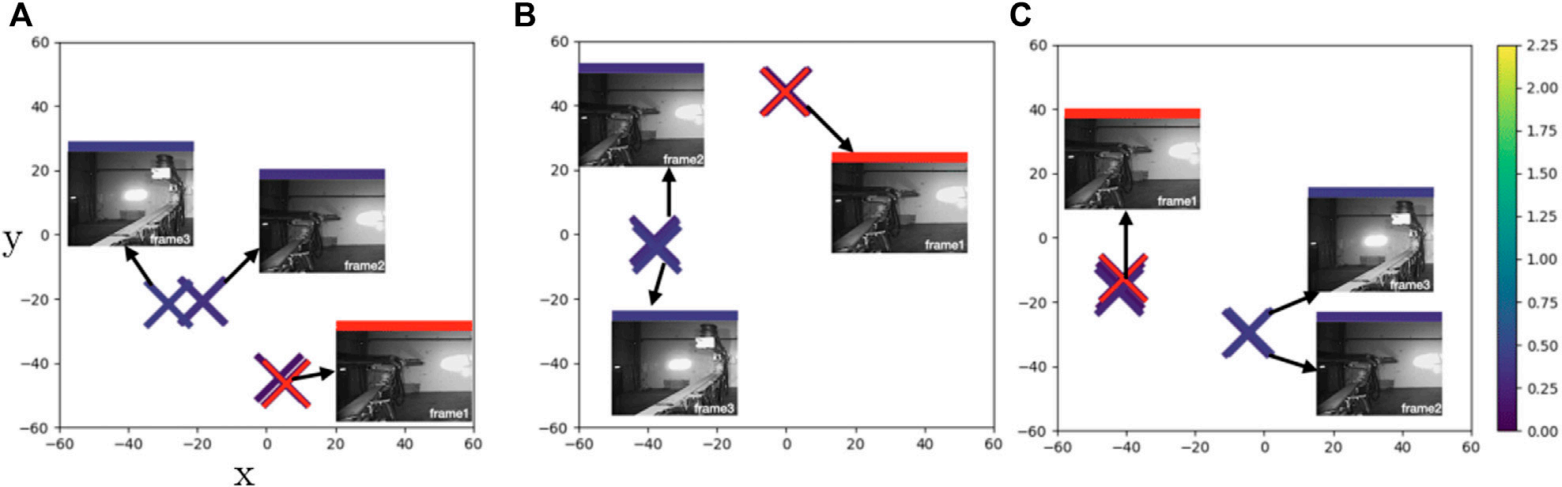
**Case4**: t-SNE plots of learned feature embeddings—**(A)** through PBS **(B)** PDA12K and **(C)** PTrip—colored with ground-truth joint states distance to a reference frame (red cross). All models fail to correct error in ground truth data and give erroneous results where similar points scattered different places.

Finally, we show another relatively simple case (**Case4**) similar to **Case1** where we mainly observe a yaw motion of the boom. For this sequence all the models fail to create a consistent result with respect to the ground truth labels (Figures 8A–C). While frame 2 and frame 1 capture almost identical end effector poses, all three models place frame 2 much closer to the dissimilar frame 3. Hence, for this sequence the feature encoders map dissimilar poses as similar, which reflects on the smoothness measure—all the models give two times larger CLA values than the ground-truth. Intuitively this makes the shape of the latent space more complex, which in turn places higher demands on the following regression network, and may be the cause of the observed high prediction errors and meager transfer capability of the three evaluated models.

### 4.4 Discussion

In our result section, we discuss several experimental results to show that our way of using data augmentation with a triplet loss increases the transferability capacity of the baseline model trained only on source domain. In these experiments, we observe an error decrease in joint state estimation in PDA12K and PTrip compared to the direct transfer baseline (PBS) in Table 1. Also in Table 2, the PTrip approach results in an improvement of roughly 30% for pose estimation compared to PBS. Moreover, our latent space analysis shows that the feature embeddings learned through PDA12K and PTrip represent the smoothness and structure of the target domain for different cases better than PBS (Figure 5 and Figure 6).

However, even though we show the improved transferability capability of our proposed method, there are limitations as well. The main limitation of our approach comes from the fact that we have not directly trained the regression task for pose estimation that is of more interest in our application. Hence, this may cause a degree of randomness to our pose estimation calculation (Figure 4), e.g., our combined metric learning and data augmentation approach (PTrip) performs better on the DISP measure (iable 2) compared to the ones in joint state estimation (Table 1). We can observe this randomness in the latent space analysis, as well. In Figure 8, for this sequence, the feature encoders of all three models map dissimilar poses as similar. As a result, we can conclude that we have a more complex shape in the latent space than the other sequences presented in Figure 5. This complex latent space places higher demands on the regression task. This causes high prediction errors and low transfer capability. Therefore, supervising the regression task directly over pose estimation can help to differentiate similar/dissimilar poses in a more accurate way in the latent space. As a result, the introduced limitation stresses the importance of more careful selection of the task for training (e.g., regression task directly over pose estimation).

## 5 Conclusion

In this paper we introduce a new transfer learning method that combines metric learning and domain-aware data augmentation. Differently from previous transfer learning methods, our approach does not use target domain data directly during training but includes target domain knowledge through source domain augmentation. We apply the method to a scenario in mining robotics that features a difficult to predict and fully capture deployment domain. We concentrate on the challenging task of estimating the joint configurations of an articulated manipulator in an unknown target domain, by only having access to labeled data from a different source domain. Our results indicate that the proposed integration of a metric learning objective and domain-aware data augmentation have a promising transfer capacity, with ≈30% improvement with respect to a model trained only on source domain data. Moreover, we qualitatively evaluate the latent space of our approach and demonstrate that the feature encoder trained results in a smooth embedding. Hence, our approach has the capacity to map images of similar manipulator configurations to close-by regions of the latent space, regardless of visual appearance. Due to the challenging transfer task however, the error obtained for joint state prediction on the target domain is still substantially higher than the ones that can be obtained by supervising the model with real in-domain data. Our future work will concentrate on further exploring the relationship between the latent space smoothness and the subsequent regression task. We also aim at devising more generic domain augmentation methods and explore adversarial approaches to generating relevant out-of-domain data.

## Data Availability

The datasets presented in this article are not readily available because Data will be made publicly available pending approval by industrial partners. Requests to access the datasets should be directed to PG, puren.guler@gmail.com.

## Footnotes

1 Epiroc AB, https://www.epirocgroup.com/.
